# Post-Partum Clinical and Patient-Reported Outcome Changes in Mothers with Multiple Sclerosis: Findings from the NAPPREMS Study

**DOI:** 10.3390/medicina60071159

**Published:** 2024-07-18

**Authors:** Dejan Jakimovski, Katelyn S. Kavak, Kara Patrick, Omid Mirmosayyeb, Svetlana P. Eckert, David Hojnacki, Bianca Weinstock-Guttman

**Affiliations:** 1 Department of Neurology, Jacobs Comprehensive MS Treatment and Research Center, Jacobs School of Medicine and Biomedical Sciences, University at Buffalo, State University of New York, Buffalo, NY 14203, USA; 2 Wynn Hospital, Mohawk Valley Health System, Utica, NY 13502, USA

**Keywords:** pregnancy, DMT, multiple sclerosis, natalizumab, anti-CD20, PRO

## Abstract

*Background and Objective*: Pregnancy in mothers with multiple sclerosis (MS) commonly results in significant changes in disease activity and changes in clinical care, including the discontinuation of disease modifying therapy (DMT). This study aimed at understanding the clinical and patient-reported outcomes (PROs) before, during and 1-year after delivery. *Materials and Methods*: A total of 30 pregnant mothers with MS were recruited as part of the study. Clinical (relapse activity and disability changes), PRO information and MRI outcomes were collected on four separate visits: one baseline visit—0–30 days post-delivery; and 3 follow-up visits at week 24, week 36 and week 52 from the baseline. PRO was assessed using a validated questionnaire called the Fatigue Scale for Motor and Cognitive Function (FSMC). The MRI scans were analyzed, and the count of new T2 lesions and/or contrast-enhancing lesions was determined. *Results*: The average time between delivery and the start of DMT was 142.5 days. Relapse activity before the pregnancy was numerically linked with the activity during the pregnancy, where up to 57.1% of the activity during pregnancy occurred in pwMS with previously active disease before conception (statistically trending with *p* = 0.073). The relapse activity after the pregnancy occurred twice as often in pwMS whose MS was clinically active before conception. All five pwMS who experienced a relapse prior to the pregnancy experienced worsening in their physical PRO domain. *Conclusions*: Pre-pregnancy activity is crucial in the screening of mothers with MS at risk for post-partum relapses, worsening of clinical disability and/or PRO measures. A post-partum MS period may benefit from the routine PRO utilization and screening for its worsening. The inflammatory activity during pregnancy was not associated with short-term disease progression.

## 1. Introduction

Multiple sclerosis (MS) is a chronic inflammatory, demyelinating, and neurodegenerative disease of the central nervous system (CNS) that is characterized by acute neurological worsening (i.e., relapses) and long-term accrual of physical and cognitive disability [1]. Recent epidemiological studies estimate more than 2.8 million people with MS worldwide, with significantly greater prevalence within the female population (2:1 or 3:1 ratio versus male) [2]. Historically, female pwMS were advised that pregnancies could potentially worsen the trajectory of their disease, a conclusion that was based on the greater occurrence of post-partum acute clinical activity [3]. On the contrary, the period of pregnancy has been generally considered to be clinically stable period, with relative “protection” of relapse activity [4].

A majority of disease-modifying therapies (DMTs) for treatment in MS could result in adverse events related to the mother’s health and are generally discontinued before the pwMS are planning for pregnancy and childbearing [5]. On the other hand, DMT withdrawal during pregnancy may as well be related to fetal side effects; however, this aspect has not been systematically investigated. Moreover, some DMTs could potentially transfer through breastfeeding and exert immunological effects on the newborn as well [5]. The increased risk of post-partum clinical activity coupled with lack of immunosuppression due to lack of DMT use can result in greater consequences of relapse-induced disability and worsening of the long-term outcomes of childbearing pwMS. Recent studies have suggested that early DMT administration of monoclonal therapy after the delivery could significantly decrease the risk of post-partum clinical activity, without significant transfer of those antibodies into the breast milk [6]. For example, the average infant dose of ocrelizumab (anti-CD20 IgG1 monoclonal antibody) transferred through breastfeeding was <1%, and the medication was nearly undetectable after 3 months, with no effect on the infant’s growth trajectory [6]. Only 2 out of 57 women experienced a post-partum clinical relapse [6]. Meta-analyses also showed that breastfeeding significantly reduces the risk for post-partum relapse risk in mothers with MS [3].

Based on this background, we aimed to explore the relapse activity before, during, and after pregnancy in pwMS and its relationship with changes in clinical, MRI, and, in particular, patient-reported outcome (PRO) measures that were recorded up to 52 weeks after delivery. Initially, the study also aimed at determining the utility of natalizumab use right after delivery in preventing post-partum activity. Due to changes in the DMT portfolios and changing therapy paradigms, the study was adapted to understand the utility of DMT use before, during, and after the pregnancy as a real-world observational study.

## 2. Materials and Methods

### 2.1. Study Population and Clinical Investigation

All study participants were recruited as part of the NAtalizumab for Prevention of Post-Partum Relapses in Patients with Multiple Sclerosis (NAPPREMS) study at the Jacobs MS Center for Treatment and Research (JMSCTR), University at Buffalo, Buffalo, NY, USA, and at the Department of Neurology in the University of Colorado, Denver, CA, USA. The inclusion criteria for the study were (1) female subjects postpartum, 0–30 days postpartum at the time of informed consent; and (2) Diagnosis of relapsing form of MS as per the 2017 McDonald criteria [7].

The exclusion criteria consisted of (1) diagnosis of primary progressive MS, (2) use of IVIG during or after the delivery, (3) significant renal or hepatic impairment (in the opinion of the investigator) or other significant diseases (e.g., cognitive impairment) that would compromise adherence and completion of the trial, and (4) history of hypersensitivity to previous exposure or presence of antibodies to natalizumab. The study consisted of 4 separate visits: one baseline visit—0–30 days post-delivery; and 3 follow-up visits at week 24, week 36, and week 52 from the baseline. If the pwMS did not complete the 52-week follow-up, the latest visit was determined as the most recent follow-up, and the time of follow-up was incorporated into the analysis.

All participants underwent full physical and neurological examinations. The disability level was assessed using the Expanded Disability Status Scale (EDSS) score [8], and the disease phenotype was determined based on the 2013 Lublin criteria [9]. The worsening in EDSS scores was determined based on an absolute increase in EDSS scores from the baseline visit to the 52-week follow-up or the most recent follow-up. Relapse was defined as the appearance of new symptoms or the return of old symptoms for a period of 24 h or more—in the absence of an infection or a change in the core body temperature. Relapses could occur when no change in EDSS was determined as well. If there was more than one event, two relapses were counted only if they were 30 or more days apart. The presence of comorbidities (hypertension, hypercholesterolemia, heart disease, asthma, emphysema, fibromyalgia, depression, anxiety disorders, diabetes, and thyroid disease), breastfeeding information, and DMT information were collected using standardized questionnaires that were administered by trained research investigators.

Information on DMT use was recorded at baseline and included data before the conception/learning of the pregnancy and over the follow-up period from the baseline post-partum up to the 52-week follow-up. The therapies were categorized based on the mode of administration; however, specific discussion regarding the use and effect of natalizumab was included.

The study was approved by the Institutional Review Board (IRB) at the University of Buffalo, State University of New York, and at the University of Colorado, all participants provided written consent forms. The study was registered in ClinicalTrials.gov with ID NCT03046251.

### 2.2. Magnetic Resonance Imaging (MRI) and MRI Outcomes

The pwMS also underwent at least two MRI examinations: the first one occurred 1–3 months postpartum (had to be before the first post-partum dose of natalizumab), and the second one was a follow-up MRI exam at the week-52 follow-up visit. The sequences utilized for the purpose of this study were T2-FLAIR and T1-weighed sequences that were acquired before and after the administration of gadolinium contrast. The MRI scan was read by a licensed and experienced neuroimager, who determined the count of new or newly enlarging T2 lesions and new T1 contrast-enhancing lesions. The new T2 lesions were based on comparisons with scans before the pregnancy.

### 2.3. Patient-Reported Outcome (PRO) Measures

The study participants also completed two patient-reported outcome (PRO) questionnaires, namely the Multiple Sclerosis Impact Scale-29 (MSIS-29) and the Fatigue Scale for Motor and Cognitive Function (FSMC). The MSIS-29 (29-item) is a psychometrically validated PRO measure that is increasingly used in trials of treatments for MS. The Rasch analysis of the previous existing MSIS-29 data led to the revision of the scale.

The FSMC is a scale that has undergone validation based on a large sample of patients and provides differential quantification and graduation of cognitive and motor fatigue The FSMC was tested against several external criteria (e.g., cognition, motivation, personality, and other fatigue scales). The item analysis and validation procedure showed that the FSMC is highly sensitive and specific in detecting fatigued MS patients. Both FSMC subscales significantly differentiated between patients and controls and demonstrated good test–retest reliability.

### 2.4. Statistical Analyses

All statistical analyses were performed using SPSS version 28.0 (IBM, Armonk, NY, USA). Data were shown as mean (standard deviation; SD) or median (interquartile ranges IQR) as appropriate. Categorical variables were compared using the chi-square test, whereas numerical variables were compared using Student’s *t*-test. The *p*-values lower than 0.05 were considered statistically significant. Regarding the sample size calculation, our initial protocol targeted *n* = 200, which would have provided 99% power for a large effect size (d = 1) or 95% power for a medium effect size (d = 0.5). That said, the current study at n = 30 and expected large effect (d = 1) results in 75% power. Despite the lower sample size, the presence of positive findings outlined hereafter further corroborates the sufficient study power.

## 3. Results

### 3.1. Demographic and Clinical Characteristics of the Study Population before Pregnancy, after Delivery, and during the 52-Week Follow-Up

The demographic characteristics of the study population are described in Table 1. The sample size for each data point is also specifically outlined in Table 1. In particular, the 30 pregnant women with MS had disease onset at a mean age of 24.0 years old (SD = 5.7), had their delivery at a mean age of 31.8 years old (SD = 5.5), and had their first study visit at a mean age of 32.3 (SD = 5.5). The majority of the pwMS were White (86.2%) and non-Hispanic (89.7%). After delivery, 51.9% of the pwMS were exclusively breastfeeding. Data on parity were only partially available, where 10 (33.3%) mothers with pwMS were primipara, and the information for the remaining 20 mothers was missing, or they were multipara.

The DMT distributions before and after the pregnancy are also shown in Table 1. A majority of pwMS were treated before pregnancy (27 out of 30): 20 on injectable and 6 on infusion DMTs. After pregnancy, 23 out of 30 pwMS (re)started treatment with either injectable DMTs (n = 13) or infusion-based therapy (n = 7, with 4 being on natalizumab and 3 on ocrelizumab). For those who restarted DMT, the mean number of days between delivery and (re)starting was 153.1 days (SD = 121.2). The majority (n = 15, 65.2%) resumed the same DMT as before pregnancy. Of the eight who switched, the changes were peg-interferon-β-1a to glatiramer acetate; interferon-β-1a to peg-interferon-β-1a, dimethyl fumarate, or natalizumab; glatiramer acetate to ocrelizumab; and natalizumab to rituximab. Additionally, two patients who did not use DMT in the year prior to pregnancy initiated dimethyl fumarate or peg-interferon-β-1a after delivery.

The clinical characteristics of the pwMS from the baseline and over the follow-up are shown in Table 2. Overall, the median EDSS scores remained fairly stable (median EDSS at baseline vs. week 52, 2.0 vs. 2.0), with only four (14.3%) pwMS experiencing EDSS worsening. The rate of relapses before, during, and after the pregnancy also remained relatively stable, with a slight decrease during the pregnancy period. During the 1 year before pregnancy, nine (30.0%) pwMS reported clinical relapses, seven (23.3%) pwMS reported clinical relapses during the pregnancy, and nine (32.1%) reported relapses after the pregnancy. In total, 8 out of 22 (36.4%) pwMS had new T2 lesions, and 4 out of 21 pwMS (19.0%) had gadolinium-enhancing lesions on their MRI examinations. From a comorbidity point of view, there was a significant presence of psychiatric diagnoses, including anxiety (eight pwMS, 26.7%) and depression (seven pwMS, 23.3%).

The mean duration to determine EDSS worsening was 45.8 weeks (12.8 months) based on a chart review for pwMS without data available at the 52-week mark. All MRI data were obtained from chart review, using the closest visit to 52 weeks post-delivery.

“Relapses prior” refers to relapses in the 12 months before pregnancy. “Relapses during” refers to relapses during pregnancy. “Relapses post” refers to the approximately 52-week period after delivery, with data partially obtained through chart review for participants with missing follow-up information. Comorbidities were reported at baseline, and the sample size of 30 reflects the assumption of “no” for those with missing data.

### 3.2. Patient-Reported Outcomes in the Study Population after Delivery

The PRO changes after the delivery are shown in Table 3. Overall, a small worsening in the overall FSMC was noted, with fatigue increasing from a median score of 51.5 at baseline to 54.0 at the most recent follow-up visit. In total, 13 (59.1%) of the pwMS experienced worsening in FSMC scores. Similar changes were seen within the MSIS Physical domain, with a small increase from a median of 27.5 at baseline to a median of 31.0 at the most recent follow-up. Twelve (54.5%) of the pwMS experienced a worsening in their MSIS Physical domain scores. Lastly, 13 (59.1%) pwMS also reported worsening in the MSIS Mental domain, with a minimal increase from 15.5 at baseline to 16.0 at the most recent follow-up.

### 3.3. Differences in Relapse Activity and Relationship with Clinical/PRO Outcomes before, during, and after the Pregnancy

The relationship of relapse activity between the timepoints is shown in Table 4. Overall, the relapse activity before the pregnancy was numerically linked with the activity during the pregnancy, where up to 57.1% of the activity during pregnancy occurred in pwMS with previously active disease before conception (statistically trending with *p* = 0.073). Similarly, the relapse activity after the pregnancy occurred twice as often in pwMS who were active before conception when compared to those that were not active before conception (44.4% of post-pregnancy relapses occurred in pwMS with relapses before pregnancy versus 21.1% of pwMS with previous activity did not experience new activity after pregnancy). Lastly, one-third (33.3%) of the post-pregnancy activity occurred in pwMS who experienced a relapse during their pregnancy.

The same investigations regarding the relationship of relapse activity before, during, and after the pregnancy and the occurrence of new MRI-based activity within 52 weeks post-delivery are shown in Table 5. The relapse activity before and during the pregnancy were not significantly associated with a greater likelihood of having either new T2-FLAIR lesions or new gadolinium-enhancing lesions. On the contrary, the relapse activity after the pregnancy was associated with a greater likelihood of having new T2-FLAIR lesions (75% vs. 23.1%, *p* = 0.02) and a numerically greater likelihood of gadolinium-enhancing lesions (75% vs. 37.5%, *p* = 0.178).

The effects of relapses before, during, and after the pregnancy on the worsening in disability and PRO outcomes over the 52-week follow-up are shown in Table 6. In contrast to the MRI outcomes, the activity before and during pregnancy was related to potential worsening in EDSS scores and PRO outcomes. In particular, all five pwMS who experienced a relapse prior to the pregnancy and had available PRO data at week 52 experienced worsening in their physical domain. The changes in disability and PRO scores occurring over all follow-up visits are outlined in Table 7 as well. In particular, the differences in MSIS Physical domain between pwMS with relapses before the pregnancy became apparent at both the second visits (3 months after delivery, 44.2 vs. 30, *p* = 0.02) and the most recent follow-up (52-week follow-up or latest available, 45.4 vs. 29.6, *p* = 0.006).

There were no differences in the 52-week follow-up PRO scores or any interim visits between pwMS with and without relapse activity during and after the pregnancy. Also, there were no differences in T2 or GAD measures in relation to any of the PROs.

### 3.4. Descriptive Clinical, MRI, and PRO Characteristics of pwMS Who Were Treated with Natalizumab before or after the Pregnancy When Compared to Anti-CD20 Monoclonal Antibodies

Of the six participants who were on infusion DMTs prior to pregnancy, two were on ocrelizumab and four were on natalizumab. Post-partum relapses were absent in the two ocrelizumab users, while two of the four natalizumab users reported post-partum relapses. One of the natalizumab users who experienced a post-partum relapse had three new T2 lesions and new GAD lesions, while the other had six new T2 lesions without new GAD lesions. Both natalizumab users who had a post-partum relapse also experienced a relapse during pregnancy, whereas only one of the ocrelizumab users reported a relapse during pregnancy.

The two natalizumab users with post-partum relapses were not exclusively breastfeeding, while the two ocrelizumab users were. Of the remaining two natalizumab users without post-partum relapses, one reported exclusive breastfeeding, and the other did not. None of the natalizumab users, regardless of relapse status, experienced worsening of their EDSS scores, which remained at 2.0 for all four. In contrast, one ocrelizumab user experienced EDSS worsening (from 1.0 to 3.0), while the other remained at 0.0.

The ocrelizumab user who had a worsened EDSS score also exhibited worsening in the MSIS Physical and Mental components and the FSMC, while the other ocrelizumab user did not have any worsening in PROs. Among the four natalizumab users, two worsened on all three PRO measures (MSIS Physical, MSIS Mental, and FSMC), with one of these individuals also experiencing post-partum relapses. One natalizumab user worsened in MSIS Mental and FSMC and also had post-partum relapses, while the remaining natalizumab user worsened only in FSMC.

After delivery, both ocrelizumab users restarted ocrelizumab therapy (timing unknown). The two natalizumab users with post-partum relapses also reinitiated natalizumab treatment at 42- and 43-months post-partum, respectively. One natalizumab user switched to rituximab (timing unknown), while information on post-partum DMT was unknown for the remaining natalizumab user.

Considering only those who reported receiving infusion DMTs after delivery, there were six pwMS: the three ocrelizumab users mentioned previously (including one who switched from glatiramer acetate (Copaxone)) and three natalizumab users. The former Copaxone user who switched to ocrelizumab did not experience EDSS worsening (EDSS = 3.5) but reported a post-partum relapse without new lesions shown on the MRI. This pwMS did not experience relapses prior to pregnancy and did not exclusively breastfeed. In terms of PROs, this person exhibited worsening in MSIS Physical but not in other aspects.

Additionally, there was one natalizumab user who did not report receiving natalizumab prior to pregnancy (switched from interferon beta-1a (Avonex)), with the initiation of natalizumab occurring in the same month as the delivery date. This pwMS did not experience any post-partum or during-pregnancy relapses but had six new T2 lesions (no change in GAD lesions) and worsening in all PROs compared to baseline. There was no change in EDSS (EDSS = 1.0).

## 4. Discussion

The findings from this study are multifold. First, the risk of activity during pregnancy and after delivery is highly correlated with the activity recorded within the 12 months before conception. Second, the worsening of disability and PRO-based outcomes after delivery is associated with the presence/amount of activity before the pregnancy and not with the activity during and after the delivery. As expected, greater activity post-partum was also associated with a higher likelihood of having new T2-FLAIR or gadolinium-enhancing lesions at the 1-year follow-up. The ramifications of these findings are discussed hereafter.

In terms of natalizumab use, there are several recent studies that have explored its use during the pregnancy and initiation right after the pregnancy. Based on the German MS pregnancy registry, the exposure to natalizumab throughout the first trimester (n = 171 and median 30.9 weeks of gestational exposure) resulted in significantly fewer relapses occurring during and in the post-partum year when compared to those who discontinued [10]. The discontinuation of natalizumab during pregnancy has been associated with up to 66.8% of pwMS experiencing relapses, where more than 16% were severe, and in 1.1%, they were life-threatening [11]. Similar to our study’s findings, up to 10% of women with MS who discontinued natalizumab during their pregnancy retain the clinically meaningful disability up to 1 year after delivery [11]. An alternative protocol that could maintain disease control during pregnancy and limit the overall dose exposure to the newborn is the natalizumab extended-interval dosing [12]. The extended-interval dosing (change from 4-week to 6- or 8-week intervals) have been shown to be equally effective in suppressing the inflammatory activity in clinical control trials [13]. From a neonatal point of view, the exposure resulted in a significantly greater likelihood of anemia and thrombocytopenia [10]. The rate of relapses in this pwMS group was significantly greater when compared to our study, with up to 62.6% of mothers who discontinued the therapy in their first trimester reporting a relapse during the pregnancy or in the first post-partum year (vs. 23.3% and 32.1% for during and post-pregnancy relapses from our study). The pattern of maintaining DMTs until becoming pregnant (particularly the use of glatiramer acetate, natalizumab, dimethyl fumarate, and anti-CD20 antibodies) has been increasing over the past decades [14]. The French nationwide study of DMT exposure during pregnancy demonstrated a 124% increase in exposed pregnancy between 2010 and 2012 and between 2019 and 2021. Moreover, a smaller proportion of mothers with pwMS discontinued their DMT during pregnancy (84% vs. 72.4% from 2012 to 2021) [14].

When compared to natalizumab, the discontinuation of anti-CD20 monoclonal antibodies before conception is associated with a significantly lower risk of disease reactivation [15]. From this perspective, several guidelines have suggested that highly active pwMS could potentially benefit from bridging to these medications before the pregnancy or remaining on natalizumab up to 34 weeks into the pregnancy [15,16]. The early start of natalizumab or fingolimod (within 3–4 months post-partum) is also an effective way of reducing the early post-partum risk of a relapse [17]. For example, 76% of pwMS who re-started on these two DMTs after pregnancy remained stable post-partum, compared to only 56% of pwMS who opted for no breastfeeding and no DMT strategy [17]. It is important to mention the indication bias, with a significantly greater % of pwMS who opt for a DMT during pregnancy having a history of active disease and previous use of second-line DMTs [17].

Based on our literature research, no pregnancy MS studies have investigated the relationship between relapse activity before, during, and after the delivery and the changes in PROs. Interestingly, we demonstrated that the early activity (prior and during pregnancy relapses) was associated with worsening in PROs, in particular, within the physical domain of the questionnaire. These findings are potentially adding additional use-case scenarios for PRO outcomes in MS, with previous publications suggesting their predictive value for disease worsening in general MS care [18]. Worse baseline reports of physical limitations or fatigue on a similar PRO questionnaire (LIFEware questionnaire) have been associated with worse long-term disease outcomes [19]. Moreover, natalizumab discontinuers also experience a significant worsening in PROs, an additional feature that may be compounded in the settings of pregnancy management [20].

There are several limitations that have to be emphasized. First, the study enrollment was substantially impacted by multiple factors. The nature of the study requires long waiting periods for pregnancies to actually occur, and not all pregnant pwMS were readily open to study enrollment. Moreover, the first year of motherhood can be demanding on pwMS, and the study experienced a significant drop-out rate over the required four follow-up study visits. Although the study initially intended to have all pregnant pwMS start on natalizumab right after the delivery (30-day window), many enrolled mothers decided to opt-out of this study requirement. All of these factors resulted in a small study sample size and diminishing number of cases available throughout the entire follow-up. Lastly, the study enrollment occurred during the entire COVID-19 pandemic, during which the enrollments in research studies and active follow-up of participants were significantly limited. While our final sample was smaller than intended due to these challenges, the detection of significant effects even with reduced statistical power underscores the potential strength of our findings. This suggests that future studies with larger sample sizes may reveal additional important outcomes and further validate our results. Lastly, the incomplete parity information prevented us from performing any additional analysis that could generalize our findings to either primipara or multipara pregnancies.

## 5. Conclusions

Pre-pregnancy activity is crucial in the screening of pwMS at risk for post-partum relapses and worsening of clinical disability and/or PRO measures. Therefore, as practitioners, we believe that the recommendation to stabilize the disease before considering pregnancy remains an important education goal. The evaluation of the post-partum period in pwMS may increasingly benefit from the routine utilization of PRO measures and screening for its worsening over follow-ups. The inflammatory activity during the pregnancy was not associated with the future short-term disease progression. Future studies should determine whether prompt initiation of highly efficacious DMT right after delivery could decrease the worsening in pwMS who had a history of active disease before the pregnancy.

## Figures and Tables

**Table 1:** The demographic and clinical characteristics of the study population.

Demographics	Mean (SD) or n (%)
Age of onset, years	24.0 (5.7)
Age at baseline visit, years	32.3 (5.5)
Age at delivery, years	31.8 (5.5)
Race	
Black or African–American	3 (10.3%)
White	25 (86.2%)
Unknown	2 (3.4%)
Ethnicity	
Hispanic or Latino	1 (3.4%)
Not Hispanic or Latino	26 (89.7%)
Unknown	3 (6.9%)
Exclusive breastfeeding at baseline	14 (51.9%)
Last DMT type/use before pregnancy	
Injectable	20 (66.7%)
Oral	1 (3.3%)
Infusion	6 (20%)
No DMT	3 (10.0%)
DMT use/type 52 weeks after delivery	
Injectable	13 (43.3%)
Oral	2 (6.7%)
Infusion	7 (23.3%)
Off label (Rituxan)	1 (3.3%)
No DMT	7 (23.3%)
Days between delivery and start DMT	153.1 (121.2)

Legend: DMT—disease-modifying therapy; MRF—most recent follow-up. Age at onset, race, and ethnicity were available in 29 pwMS.

**Table 2:** Clinical characteristics of the study population and study outcomes.

Variables	Mean (SD)—Median (IQR) or n (%)
**EDSS**	
EDSS baseline (n = 29)	1.6 (0.9)–2.0 (1.0–2.0)
EDSS visit 1 (n = 24)	1.7 (0.9)–1.5 (1.0–2.0)
EDSS visit 2 (n = 12)	1.4 (0.9)–1.3 (1.0–2.0)
EDSS visit 3 (n = 5)	1.6 (1.2)–1.5 (0.5–2.8)
EDSS week 52 (n = 29)	1.8 (0.9)–2.0 (1.0–2.5)
EDSS worsening (n = 28) (52 weeks vs. baseline)	4 (14.3%)
**MRI**	
New T2 lesions (n = 22)	8 (36.4%)
New GAD lesions (n = 21)	4 (19.0%)
**Relapses**	
Relapses prior conception (n = 30)	9 (30.0%)
Relapses during pregnancy (n = 30)	7 (23.3%)
Relapses post-partum (n = 28)	9 (32.1%)
**Comorbidities (n = 30)**	
Hypertension	1 (3.3%)
Asthma	1 (3.3%)
Depression	7 (23.3%)
Anxiety disorders	8 (26.7%)
Thyroid disease	1 (3.3%)

Legend: EDSS–Expanded Disability Status Scale; MRI—Magnetic Resonance Imaging; GAD—gadolinium-enhancing.

**Table 3:** Patient-reported outcomes over the 52-week follow-up after delivery.

PRO	Mean (SD)—Median (IQR) or n (%)
**FSMC**	
FSMC visit 1 (n = 28)	51.8 (16.7)–51.5 (37.3–62.0)
FSMC2 visit 2 (n = 22)	52.9 (23.7)–53.0 (33.5–71.5)
FSMC3 visit 3 (n = 6)	59.3 (26.2)–54.5 (37.8–89.0)
FSMC MRF (n = 22)	53.8 (22.9)–54.0 (34.3–71.5)
FSMC worsening (MRF vs. visit 1)	13 (59.1%)
**MSIS Physical**	
MSIS Physical visit 1 (n = 28)	31.7 (11.4)–27.5 (23.3–37.8)
MSIS Physical visit 2 (n = 22)	33.2 (12.4)–30.5 (22.5–42.3)
MSIS Physical visit 3 (n = 6)	36.4 (9.5)–35.0 (31.0–44.0)
MSIS Physical MRF (n = 22)	33.2 (11.9)–31.0 (23.0–43.0)
MSIS Physical worsening (MRF vs. visit 1)	12 (54.5%)
**MSIS Mental**	
MSIS Mental visit 1 (n = 28)	16.7 (5.4)–15.5 (13.3–19.8)
MSIS Mental visit 2 (n = 22)	16.9 (6.6)–15.5 (11.8–21.0)
MSIS Mental visit 3 (n = 6)	21.6 (7.5)–20.0 (16.0–26.0)
MSIS Mental MRF (n = 22)	17.1 (6.8)–16.0 (11.8–20.3)
MSIS Mental worsening (MRF vs. visit 1)	13 (59.1%)

Legend: PRO—patient-reported outcome; FSMC—Fatigue Scale for Motor and Cognitive Functions; MSIS—Multiple Sclerosis Impact Scale, higher scores indicate worse outcome. Most recent follow-up visit—visit 3 if data available, otherwise visit 2. Worsening = increase in score between most recent follow-up and visit 1.

**Table 4:** Pregnancy relapse timepoints and subsequent relapse rates.

Relapses	Timepoint	Relapses	No Relapses	*p*-Value
Relapses prior (n = 9)	During pregnancy	4/7 (57.1%)	5/18 (21.7%)	*0.073*
Relapses prior (n = 8)	Post pregnancy	4/9 (44.4%)	4/19 (21.1%)	0.201
Relapses during (n = 9)	Post pregnancy	3/9 (33.3%)	3/19 (15.8%)	0.291

Legend: *p*-values are from chi-square tests comparing the frequency of relapses occurring during pregnancy or post-pregnancy based on relapses in the previous 1-year timepoint. *p*-values lower than 0.05 were considered statistically significant and shown in bold, whereas *p*-values < 0.1 as statistically trending and shown in italics.

**Table 5:** Frequency of new T2 and gadolinium-enhancing (GAD) lesions based on relapses occurring 1 year prior to, during, or after pregnancy.

Variable	Lesion Type	New Lesions	No New Lesions	*p*-Value
Relapses prior (n = 8)	T2-FLAIR	3/8 (37.5%)	5/14 (35.7%)	0.933
Relapses prior (n = 8)	GAD	2/4 (50.0%)	6/17 (35.3%)	0.586
Relapses during (n = 6)	T2-FLAIR	2/8 (25.0%)	4/14 (28.6%)	0.856
Relapses during (n = 6)	GAD	1/4 (25.0%)	5/12 (29.4%)	0.861
Relapses post (n = 9)	T2-FLAIR	6/8 (75.0%)	3/13 (23.1%)	**0.020**
Relapses post (n = 9)	GAD	3/4 (75.0%)	6/16 (37.5%)	0.178

Legend: T2-FLAIR—T2-Fluid Attenuated Inversion Recovery; GAD—gadolinium-enhancing lesions. The *p*-values are from chi-square tests comparing lesion rates between those with and without relapses for each timepoint/outcome. A *p*-value lower than 0.05 was considered statistically significant and is shown in bold.

**Table 6:** Frequencies show the proportion of participants with worsening on the Expanded Disability Status Scale (EDSS), Fatigue Scale for Motor and Cognitive Functions (FSMC), and Multiple Sclerosis Impact Scale (MSIS) Physical and Mental components, stratified by relapses that occurred prior to, during, or after pregnancy.

Variable	Category	Worsened	Not Worsened	*p*-Value
Relapses prior (n = 9)	EDSS	2/4 (50.0%)	7/24 (29.2%)	0.409
Relapses prior (n = 5)	FSMC	2/13 (15.4%)	3/9 (33.3%)	0.323
Relapses prior (n = 5)	MSIS Physical	5/12 (41.7%)	0/10 (0%)	**0.020**
Relapses prior (n = 5)	MSIS Mental	3/13 (23.1%)	2/9 (22.2%)	0.962
Relapses during (n = 7)	EDSS	1/4 (25.0%)	6/24 (25.0%)	1.000
Relapses during (n = 5)	FSMC	4/13 (30.8%)	1/9 (11.1%)	0.279
Relapses during (n = 5)	MSIS Physical	2/12 (16.7%)	3/10 (30.0%)	0.457
Relapses during (n = 5)	MSIS Mental	3/13 (23.1%)	2/9 (22.2%)	0.962
Relapses post (n = 9)	EDSS	0/4 (0%)	9/23 (39.1%)	0.125
Relapses post (n = 5)	FSMC	2/13 (15.4%)	3/9 (33.3%)	0.323
Relapses post (n = 5)	MSIS Physical	2/12 (16.7%)	3/10 (30%)	0.457
Relapses post (n = 5)	MSIS Mental	2/13 (15.4%)	3/9 (33.3%)	0.323

Legend: PRO—patient-reported outcomes; FSMC—Fatigue Scale for Motor and Cognitive Functions; MSIS—Multiple Sclerosis Impact Scale. The *p*-values are from chi-square tests comparing worsening rates between those with and without relapses for each timepoint/outcome. A *p*-value lower than 0.05 was considered statistically significant and is shown in bold.

**Table 7:** Mean scores on the Expanded Disability Status Scale (EDSS), Fatigue Scale for Motor and Cognitive Functions (FSMC), and Multiple Sclerosis Impact Scale (MSIS)’s Physical and Mental components, stratified by relapses that occurred in the 12-months prior to pregnancy.

Variable	Sample Size	Relapses’ Prior Mean (SD)	Sample Size	No Relapses’ Prior Mean (SD)	*p*-Value
**EDSS**					
EDSS Baseline	9	1.9 (1.0)	20	1.5 (0.8)	0.207
EDSS Visit 1	6	1.8 (1.1)	18	1.6 (0.9)	0.801
EDSS Visit 2	4	1.9 (1.3)	8	1.1 (0.6)	0.189
EDSS Visit 3	3	1.8 (1.6)	2	1.3 (0.4)	0.663
EDSS Closest to Week 52	9	2.2 (0.8)	20	1.6 (0.9)	0.107
**FSMC**					
Sum FSMC Visit 1	9	55.4 (13.6)	19	50.1 (18.1)	0.436
Sum FSMC Visit 2	5	65.0 (13.2)	17	49.4 (25.2)	0.202
Sum FSMC Visit 3	1	53.0 (-)	5	60.6 (29.0)	0.823
FSMC MRF	5	61.8 (13.9)	17	51.4 (24.7)	0.385
**MSIS Physical**					
Sum MSIS Physical Visit 1	9	33.9 (12.3)	19	30.6 (11.1)	0.489
Sum MSIS Physical Visit 2	5	44.2 (13.4)	17	30.0 (10.4)	**0.020**
Sum MSIS Physical Visit 3	1	44.0 (-)	6	35.2 (9.7)	0.440
Sum MSIS Physical MRF	5	45.4 (13.0)	17	29.6 (9.1)	**0.006**
**MSIS Mental**					
Sum MSIS Mental Visit 1	9	18.1 (5.2)	19	16.1 (5.6)	0.359
Sum MSIS Mental Visit 2	5	20.2 (4.0)	17	15.9 (7.0)	0.211
Sum MSIS Mental Visit 3	1	21.0 (-)	6	21.7 (8.3)	0.943
Sum MSIS Mental MRF	5	20.6 (3.9)	17	16.1 (7.2)	0.196

Legend: PRO—patient-reported outcomes; FSMC—Fatigue Scale for Motor and Cognitive Functions; MSIS—Multiple Sclerosis Impact Scale. The *p*-values from independent *t*-tests compare scores between those with and without prior relapses. The *p*-values lower than 0.05 were considered statistically significant and are shown in bold.

## Data Availability

The data presented in this study are available upon a reasonable request from the corresponding authors (D.J. or B.W.-G.).
